# Protein Kinase A Distribution Differentiates Human Glioblastoma from Brain Tissue

**DOI:** 10.3390/cancers10010002

**Published:** 2017-12-21

**Authors:** Carla Mucignat-Caretta, Luca Denaro, Domenico D’Avella, Antonio Caretta

**Affiliations:** 1 Department of Molecular Medicine, University of Padova, Padova 35131, Italy; 2 Biostructures and Biosystems National Institute, Rome 00136, Italy; antonio.caretta@unipr.it; 3 Department of Neuroscience, University of Padova, Padova 35131, Italy; luca.denaro@unipd.it (L.D.); domenico.davella@unipd.it (D.D.); 4 Department of Food and Drug, University of Parma, Parma 43121, Italy

**Keywords:** glioblastoma, cAMP, protein kinase A, diagnosis

## Abstract

Brain tumor glioblastoma has no clear molecular signature and there is no effective therapy. In rodents, the intracellular distribution of the cyclic AMP (cAMP)-dependent protein kinase (Protein kinase A, PKA) R2Alpha subunit was previously shown to differentiate tumor cells from healthy brain cells. Now, we aim to validate this observation in human tumors. The distribution of regulatory (R1 and R2) and catalytic subunits of PKA was examined via immunohistochemistry and Western blot in primary cell cultures and biopsies from 11 glioblastoma patients. Data were compared with information obtained from 17 other different tumor samples. The R1 subunit was clearly detectable only in some samples. The catalytic subunit was variably distributed in the different tumors. Similar to rodent tumors, all human glioblastoma specimens showed perinuclear R2 distribution in the Golgi area, while it was undetectable outside the tumor. To test the effect of targeting PKA as a therapeutic strategy, the intracellular cyclic AMP concentration was modulated with different agents in four human glioblastoma cell lines. A significant increase in cell death was detected after increasing cAMP levels or modulating PKA activity. These data raise the possibility of targeting the PKA intracellular pathway for the development of diagnostic and/or therapeutic tools for human glioblastoma.

## 1. Introduction

Glial tumors of the brain are classified into four grades according to World Health Organization staging. Glioblastoma multiforme (GBM) is the most malignant (grade IV) glial tumor, with a dismal prognosis, despite recent therapeutic advances. GBM is highly invasive and recurs in nearly 90% of cases at the original site [1]. Multiple intracellular signaling pathways modulate events during cell proliferation. Cyclic AMP (cAMP) and cAMP-dependent protein kinases, also named protein kinases A (PKAs) play various roles in this process [2]. The quantity of cAMP varies during the cell cycle. Low cAMP levels are detected at mitosis, while higher levels are present in G1 and early S; on the other hand, PKA activation influences the cell cycle, because PKA phosphorylates the macromolecular complexes responsible for the destruction of mitotic cyclins and separation of the sister chromatids at anaphase–metaphase transition [3]. The cAMP-mediated pathway is also linked to Ras protein activation through multiple steps: PKA activation inhibits cell proliferation, with negative feedback through the phosphorylation of phosphodiesterases that ultimately lowers cAMP concentration [4]. Data from gene expression profiling in high-grade gliomas reveal the upregulation of several proteins of the cAMP pathway, including A-kinase anchoring proteins (AKAPs) that dock PKA at precise intracellular sites [5], and phosphodiesterases [6], while the catalytic subunit of PKA is reduced [7].

PKA are tetramers of two regulatory and two catalytic subunits, present in every cell where they regulate several processes [8]. When the regulatory subunits bind two molecules of cAMP, the catalytic subunits are released and in turn phosphorylate their target proteins. Their target is addressed by PKA intracellular localization, obtained through anchoring at specific sites in macromolecular complexes, and through the expression of specific subunits. There are four regulatory (R1A, R1B, R2A, R2B) and three catalytic subunits (CA, CB, CG), that may be combined to obtain enzymes with different biochemical properties [9]. During both physiological and pathological states, the components of the PKA holoenzymes as well as their localizations may change [10,11]. In the brain of tetrapods we described the variations that take place during ontogenesis in the docked, insoluble PKA regulatory isoforms: R1A is only found in neurons of different brain areas, while R1B is restricted to few neuron types [12,13]. The highly expressed R2B is present in both neurons and glia, while R2A appears restricted to the ependymal cells [14]. In rodent and human glioma cells we observed a peculiar enrichment in R2A clusters, located at the Golgi apparatus, which are not present in healthy astrocytes within the brain [15]. As reported by other groups, we confirmed that activating the PKA system results in rodent glioma cell apoptosis [16]. Moreover, in human medulloblastoma specimens, the distribution of PKA regulatory subunits was different from that observed in glioblastoma models [17]. These observations point to PKA as a possible molecular target for tumor diagnosis and therapy [18]. Therefore, we sought to extend our data by testing the presence and distribution of PKA subunits in a series of both high- and low-grade human glioma specimens. The data presented here show that in human brain tumor specimens (Supplementary Table S1), the presence of R2 varied according to the tumor type. Moreover, targeting of PKA activity in four human glioblastoma cell lines, harboring different mutations, resulted in cell death, suggesting that selective activation of PKA may be an additional strategy for tumor therapy.

## 2. Results

### 2.1. PKA Gene Expression

**Figure 1:**
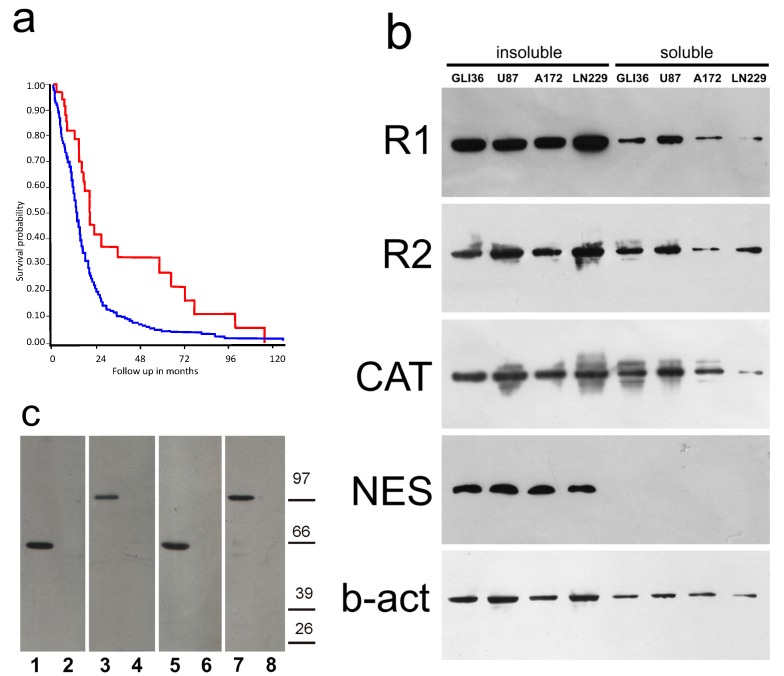
(**a**) Survival of glioblastoma multiforme (GBM) patients, clustered according to *PKAR2A* gene expression. Data from the the Cancer Genome Atlas (TCGA) database. Patients showing the highest *PKAR2A* gene expression (*n* = 469) had lower survival (lower blue line), as compared to patients showing a lower expression (highest red line, *n* = 35). (**b**) Western blot on insoluble (first four lanes) and soluble (four lanes to the right) fractions of Gli36, U87, A172, and LN229 human glioblastoma cells. From the top, they were reacted with anti-R1, R2, cAMP-dependent protein kinase (PKA) catalytic subunit (CAT), nestin (NES), and beta-actin (b-act). R1, R2, and catalytic subunits were present in both soluble and insoluble fractions. All cell lines were nestin-positive. (**c**) Western blot on insoluble (lanes 1, 3) and soluble (lanes 2, 4) fractions of human glioblastoma biopsy (case 6), and on insoluble (lanes 5, 7) and soluble (lanes 6, 8) fractions of the primary culture derived from the same biopsy, reacted with anti-R2 (lanes 1, 2, 5, 6) and CD133 (lanes 3, 4, 7, 8). Molecular weight markers are indicated on the right. R2 was present the insoluble fractions of both the biopsy and primary culture derived from it. The samples were CD133-positive.

Inspection of data from the The Cancer Genome Atlas (TCGA) database was performed on the expression signals for the regulatory PKA subunits in 540 cases of GBM, compared to non-tumor brain samples from the Genotype Tissue Expression (GTEX) study (Figure S1a). R2A appeared to be the only PKA subunit clearly upregulated in GBM, suggesting an increase in the expression of R2A in GBM. When patients were clustered according to the expression level of R2A, it was apparent that patients with higher R2A expression had shorter survival time compared to patients with lower R2A expression (chi-squared 10.81, *df* = 1, *p* < 0.001 (Figure 1a)). These data are supported also by inspection of a different database (see Figure S1b). Apparently, GBM samples present a R1/R2 balance that is different from that found in healthy tissue.

### 2.2. PKA Distribution in Glioma Cell Cultures

The presence of the catalytic and regulatory subunits of PKA was examined by Western blot in four human GBM cell lines, fractionated into soluble and membrane-bound fractions. All the four cell lines were positive for nestin (Figure 1b, NES). Both R1, R2, and the catalytic (CAT) subunits were present in the soluble and in the insoluble fractions, with both R1 and R2 mostly concentrated in the insoluble fraction (Figure 1b). These data demonstrate a different compartmentalization of the various subunits in glioblastoma cell lines.

The insoluble and soluble fractions of human glioblastoma biopsies were compared to the corresponding fractions of the primary culture derived from the same biopsy, showing the same pattern of reactivity for both R2 and CD133 (Figure 1c, case 6).

### 2.3. Altering PKA Activity Results in Cell Death

**Figure 2:**
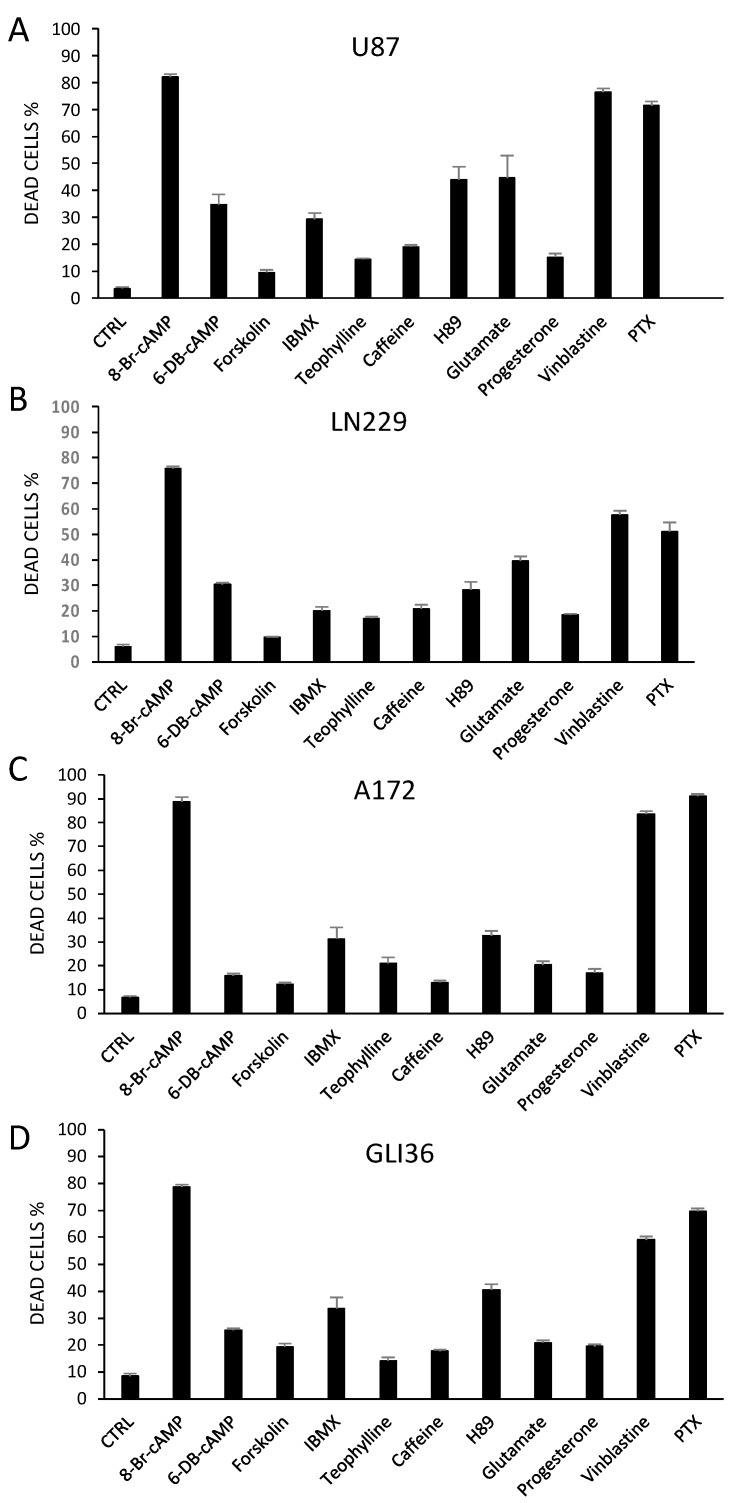
Human glioblastoma cell lines were incubated with the indicated agents for 24 h. (**A**) U87 cell line; (**B**) LN229 cell line; (**C**) A172 cell line; (**D**) GLI36 cell line. The percentage of dead cells was calculated using a total of 2400 cells for each treatment, in each cell line. Mean + S.E. Each treatment in each cell line differs from control (CTRL vs. drug, *t*-test, *p* < 0.05). After 24 h, a significant increase in cell death was detected in cells treated with agents directly acting on PKA, as well as with pro-apoptotic or antiblastic agents (glutamate, or vinblastine and paclitaxel, respectively). CTRL: control; cAMP: cyclic AMP; IBMX: isobutylmethylxanthine; PTX: paclitaxel.

**Figure 3:**
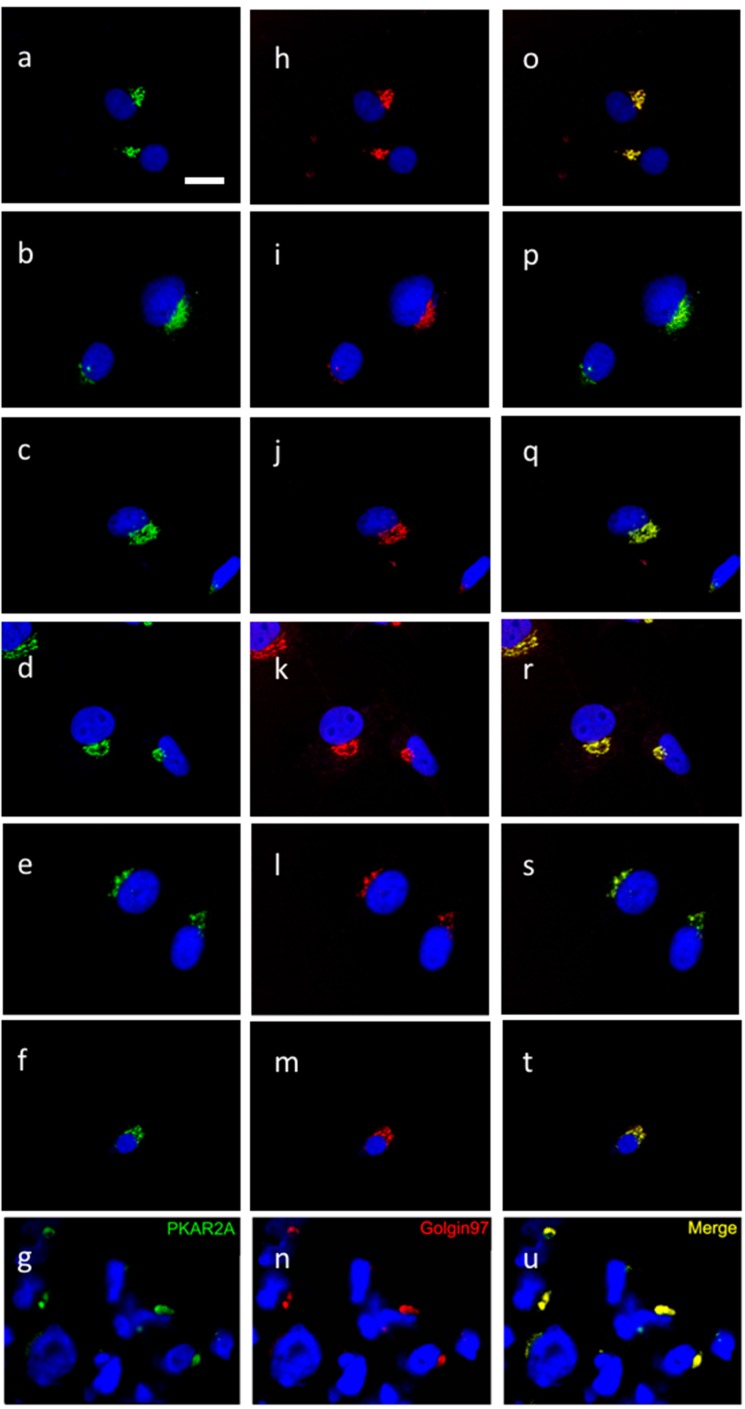
The distribution of PKA R2 in confocal images from: U87 (**a**,**h**,**o**), A172 (**b**,**i**,**p**), LN229 (**c**,**j**,**q**), and GLI36 (**d**,**k**,**r**) human cell lines, as well as from two primary cell cultures—from patients 6 (**e**,**l**,**s**) and 9 (**f**,**m**,**t**), and from GBM tissue (patient 9, **g**,**n**,**u**). R2 immunoreactivity (green, **a**–**g**) was detected in the perinuclear zone and co-localized with Golgin97 (red, **h**–**n**) as shown by the merging signals (**o**–**u**); DRAQ5 nuclear staining is shown in blue. Bar = 10 μm.

**Figure 4:**
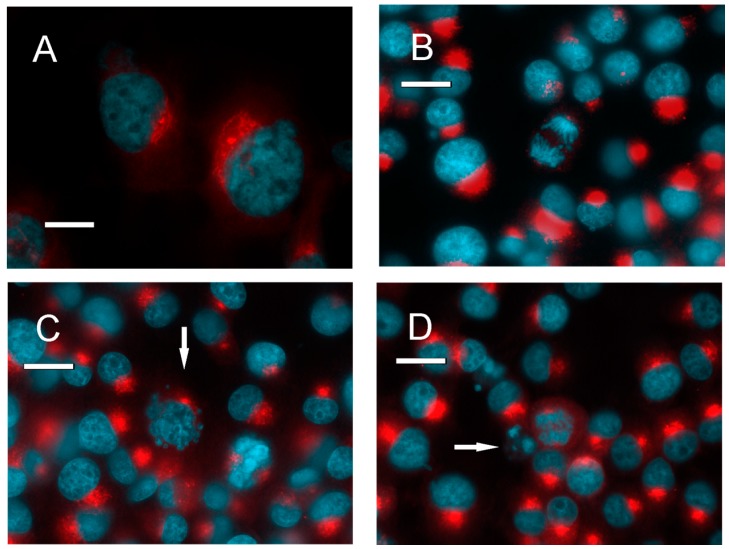
(**A**–**D**) The distribution of PKA regulatory subunit R2 changes during the cell cycle. (**A**) GLI36 cells: R2 immunoreactivity (red), bis-benzimide nuclear staining (blue). (**B**) GL261 cells during mitosis: R2 (red), bis-benzimide (blue). (**C**) GL261 cells: one apoptotic cell can be detected (arrow). (**D**) GL261 cells: one apoptotic cell can be detected (arrow). Bar = 10 μm.

**Figure 5:**
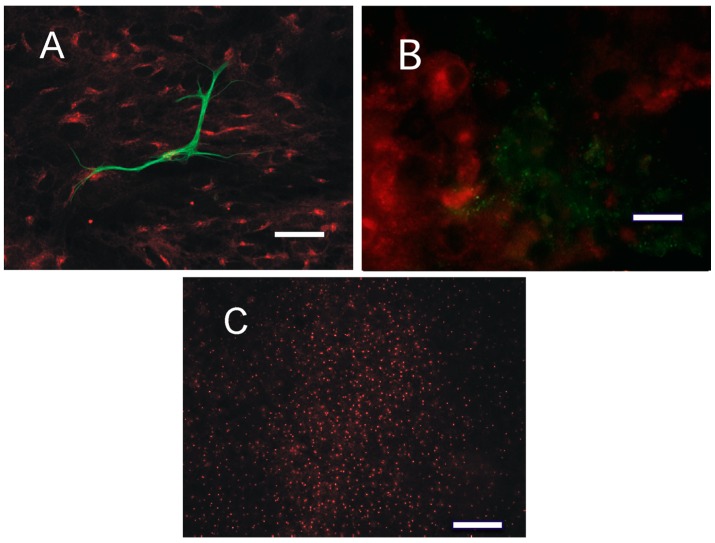
(**A**) Primary culture from glial cells: R2 (red) and glial fibrillary acidic protein (GFAP; green). Only one, presumably more differentiated cell expresses GFAP, while R2 is present in all cells. In the GFAP-positive cell, R2 is present but is less intense than in the other cells. (**B**,**C**) Representative distribution of PKA R1 and the catalytic subunit in glioblastoma samples. (**B**) R1 distribution (red) consists of small clusters of dots (case 4) clearly separated from R2. (**C**) Catalytic distribution (red) consists of a series of isolated dots (case 3). Bar = 15 μm in (**B**); bar = 50 μm in (**A**,**C**).

To establish whether the cAMP-activated intracellular signaling pathway could be a target for glioblastoma pharmacological treatment, we tested a panel of cAMP/PKA-affecting agents, and examined their effect on survival of four human GBM cell lines. Interference with the cAMP–PKA pathway induced cell death (Figure 2). A significant increase in the percentage of dead cells was detected after 24 h in all cell lines with agents that acted directly on PKA, either by activating (8Br-cAMP, 6-dibutiryl-cAMP) or inhibiting it (H89), as well as with apoptosis-inducing (glutamate) and antiblastic agents (vinblastine and paclitaxel). Forskolin and the xanthines, which act through the cAMP-producing and degrading enzymes to increase cAMP production in the cell, also showed an overall effect of increasing cell mortality. Immunocytochemistry showed that R2 was present in the all four human GBM cell lines (Figure 3), as was already shown for mouse and rat GBM cells [15], and colocalized with the Golgi apparatus. The distribution of R2 was apparently modified during the cell cycle (Figure 4A,B,D). It was still detectable in the first stages of apoptosis (Figure 4C), but was no longer visible in dead cells (Figure 4D, arrow). A similar distribution could be seen also in primary cultures of glial cells (Figure 5A), in which the presence of R2 appeared to be negatively correlated with glial fibrillary acidic protein (GFAP) expression, since it became less visible as long as GFAP signal increased. When primary cell cultures were obtained from normal brain tissue, only some cells were positive for R2 (Figure S2a), while all the cells derived from GBM specimens were positive (Figure S2b–e). The same cells showed a diffuse presence of R1 in the cytoplasm (Figure S2f).

### 2.4. Distribution of PKA in the Tumor Tissue

The in vitro data showed that PKA levels could vary in tumor cells, and that PKA activity could influence cell survival. The next step was to examine the complex brain tumor tissue for the distribution of PKA subunits. Immunofluorescence on the GBM specimens showed that R1 was present in isolated clusters throughout the tissue, in variable grades: under the present conditions, some GBM specimens were not labeled at all, while others presented clusters of R1 in some areas of the tumors (Figure 5B, Supplementary Figure S3a,b and Supplementary Table S2). A dotted distribution was detected also for the PKA catalytic subunit (Figure 5C and Figure S3c).

**Figure 6:**
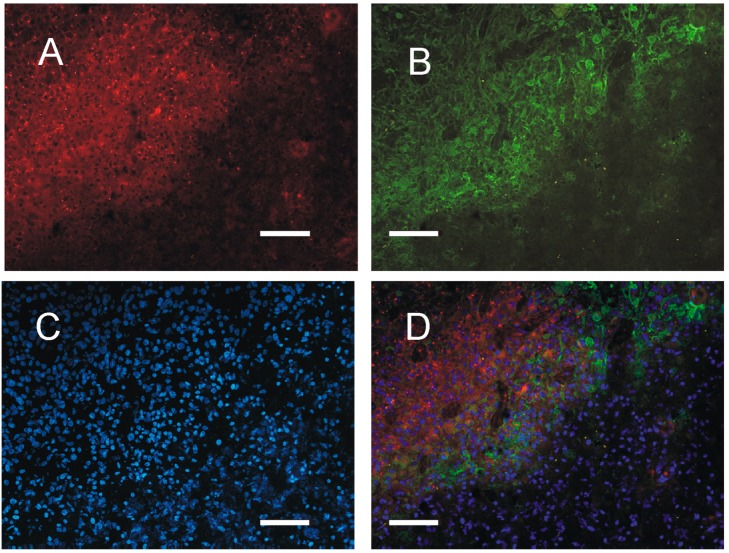
Representative distribution of PKA regulatory subunit R2 in a glioblastoma sample (case 4). The same section was immunostained for R2 and GFAP and counterstained with bis-benzimide. (**A**) R2 (red) is present in the whole tumor area (upper left) and also in some cells in the parenchyma surrounding the tumor (lower right); (**B**) GFAP (green) is present in some cells, mainly concentrated at the tumor border; (**C**) bis-benzimide nuclear counterstaining (blue) stains all nuclei; (**D**) merging of (**A**–**C**) shows that the identification of tumor area is best obtained with R2. Also in the human GBM tissue, the cells heavily labeled with GFAP present a less intense R2 signal. Bar = 50 μm.

At the cellular level, PKA R2 subunit distribution was reminiscent of that observed in cell cultures, and co-localized with a Golgi marker (Figure 3u). The same distribution was retained also in primary cultures derived from GBM (Figure 3s,t). In GBM tissue, R2 was detected in the tumor cells, so that it was possible to clearly distinguish the tumor from the adjacent tissue. Meanwhile, other markers (for example, GFAP) were not present in the overall tumor tissue (Figure 6 and Supplementary Figure S3d–f). Cases 3 and 5 were examined at relapse also, at two years and one year after surgery respectively, and after various cycles of chemotherapy and radiotherapy. In these tissues the immunolabeling was almost undetectable due to the high particulate reddish autofluorescence that was present throughout the tissue, most probably due to the effects of radiotherapy on the tissue (data not shown). When other tumors were examined, R2 appeared less evenly distributed (Supplementary Figure S4), yet most cells were positive. When present, R2 was positive in areas in which non-autofluorescent cells were present. Since yellowish autofluorescent material (mainly lipofuscins) is accumulated by long-lived cells and is easily detectable in older healthy brains with both red and green fluorescence filters, it is conceivable that areas completely devoid of autofluorescence are constituted by younger cells, as they are newly differentiated from the tumor. Both PKA R1 and catalytic subunits showed a distribution similar to GBM (Supplementary Figure S5), when present (Supplementary Table S2). Various brain metastases from different tumors were also evaluated: all of them were autofluorescent, they were positive for R2 but presented a different localization from GBM samples. They were barely reactive for R1 and the catalytic subunit, except in the two pulmonary carcinoma metastases that were clearly positive for the catalytic subunit.

## 3. Discussion

To date, the treatment options for malignant gliomas are disappointing. Interference with intracellular signaling pathways that modulate cell proliferation may be a viable target. GBM presents neither clear molecular signatures nor peculiar genetic alterations [19], and therefore it is a candidate for exploring intracellular signaling anomalies with the aim of finding new diagnostic and therapeutic targets.

The anomalies found in GBM are diverse and numerous. Among the most frequent are the gain of chromosome 7, in which the *PRKAR2B* gene resides, and alterations in chromosome 17, which hosts the *PRKAR1A* gene. In this way, the balance of PKA R1 to R2 subunits is altered [20]. This balance is important for the maintenance of cell homeostasis, and its disruption may drive cells to the tumorigenic phenotype.

Interactions among different intracellular pathways ultimately determine the cellular response. Hence, even minor changes in the organization of a single pathway may have large and unexpected consequences on cell function. Moreover, activation of the signaling pathway, that controls cellular functions critical to maintaining cancer characteristics, may be reversed by changing intracellular conditions. For example, an increase in cAMP concentration may shift the activation pattern from R1 (high affinity) to R2 (low affinity) subunits, in turn affecting their downstream effectors [21]. It has long been recognized that PKA is 10 times more abundant in GBM than in the normal brain [22], that glioma cells contain protein kinase A type 2, and that after cAMP stimulation, the catalytic subunits redistribute to particulate fractions [23]. Moreover, PKA imbalance has been linked to tumorigenesis [24]. We showed in murine GBM models that the PKA R2 subunit is concentrated in the Golgi area, and that interference with PKA activity may lead mice glioblastoma cells to apoptotic death [15]. The present data extend these observations to human glioblastoma cell lines and to human tissue samples. Interrogation of databases also supported an increase in R2A expression in GBM specimens, and poor survival of patients showing the highest R2A expression, in agreement with the dysregulation of PKA reported in different types of cancer [25,26].

In four human glioma cell lines, R2 and the catalytic subunit may be found in soluble and particulate cell fractions. In contrast to what is seen in normal brain primary cultures, both the glioma cell line and GBM primary culture present a diffuse R1 cytoplasmic labeling and a striking hotspot of R2 co-localized with the Golgi marker, similar to what was observed in rat and mouse GBM cell lines [15]. Therefore, R2 clusters in the Golgi may be considered a peculiar trait common to GBM cells.

Immunohistochemistry on GBM biopsies revealed that R1 is scarcely present (in isolated hotspots) in some specimens. Even the catalytic subunit is present in discrete clusters. The most striking feature of GBM samples is however the consistent R2 immunoreactivity in the tumor cells, located at the Golgi, an association that was previously observed in different cells [27]. R2 distribution in GBM is different from medulloblastoma [17] and resembles the distribution observed in the mouse and rat glioma models [9]. This is relevant, since the expression of some proteins has been recently shown to change when culturing GBM, as compared to the tissue sample [28]. In any case, R2 distribution is maintained when establishing primary cultures from GBM samples. In contrast to R2, GFAP is present in only some but not all tumor cells. Exploration of different tumors of the central nervous system showed that R2 is less evenly distributed. When present, R2 is never detected in autofluorescent cells, suggesting that the labeled cells are younger. On the other hand, PKA catalytic and R1 subunits are distributed similarly to GBM.

These data suggest that R2 immunoreactivity may be exploited for diagnostic purposes in GBM samples. However, it should be considered that after irradiation and chemotherapy, the tissue was too autofluorescent to be examined with reliability. The amount of intracellular cAMP varies during cell cycle in malignant gliomas, being higher in G0/G1 and lower at mitosis: cAMP arrests the cell cycle and induces differentiation and apoptosis, possibly by altering the rate of PKA subunit degradation [16], in this way linking PKA to glioma growth [29]. On average, GBM cells show lower cAMP and adenylate cyclase activity compared to healthy brain tissue [30]. The data on cell lines support the notion that PKA dynamically relocates during the cell cycle, possibly influencing the phosphorylation state of target proteins in accordance with data suggesting that an increase in the intracellular levels of cAMP, induced by different stimuli, triggers a change in cell morphology and differentiation, while their proliferation is inhibited [31,32,33]. It was also suggested that alterations of the cAMP pathway may initiate the immortalization phase of carcinogenesis [33]. We observed that increasing cAMP levels with different agents instructs GBM cells to cell death. All cell lines responded similarly to these agents, despite the fact that they harbored different mutations, thus suggesting that the cAMP pathway may alter cell survival independently from the mutated genes.

Of note, the effects of cAMP on glioma cells are reversible, and a few cells are not sensitive to cAMP action [34]. Therefore, appropriate schedules of administration should be designed for administration of agents acting via an increase in cAMP. The effects of PKA on glioma cells are also mediated by the modification of transcription. cAMP-induced differentiation results in the reduction of a number of proteins, including heat shock protein 70 [35] and c-jun [36]. On the other hand, the transcription of other proteins is enhanced, for example creatine kinase [37], S-100 and the immediate-early response gene *IEX-1* [38], and GFAP [39,40]. GFAP is a marker for astrocytic differentiation and we observed that in GBM primary cultures, R2 immunoreactivity was stronger in the absence of GFAP, and became fainter as long as the GFAP marker became stronger, further supporting the idea that R2 expression is linked to a less differentiated cell phenotype. 

Conceivably, the cAMP system is not a suitable pharmacological target because of its universal distribution and its implication in almost every cellular process, in both normal and pathological cells. Therefore, a more selective targeting of GBM-specific features should be exploited. If generic PKA acting agents are not specific enough and may induce off-target unwanted effects, other strategies could involve the targeting of pathways that use PKA in GBM-specific processes. For example, it could be interesting to act on different pathways that use PKA as an effector. Various dopamine receptors have been implicated in GBM stem cell survival and proliferation [41]. They act similarly to type 2 dopamine receptors by inhibiting adenylate cyclase to modulate the autophagy–lysosomal pathway, which may be linked to the Golgi apparatus activity. Moreover, the inflammatory pathway acting through cyclooxygenase-2 and its main product prostaglandin E2 is also involved in GBM development [42]. Interestingly, the effects of prostaglandin E2 in GBM are mediated through the PKA R2 subunit, which makes this protein an appealing target for GBM treatment [43].

Therefore, the activation of PKA in glioma cells induces several processes, that ultimately lead to differentiation. If the presence of R2A is higher in GBM than in healthy brain tissue, then it would be desirable to reduce it, with the aim of sponsoring the expression of the other PKA subunits that are necessarily induced for healthy cell survival.

## 4. Materials and Methods

### 4.1. Analysis of Gene Expression

The TCGA database was searched to obtain gene expression data for PKA regulatory subunit coding genes (*PRKARIIA*, *PRKARIIB*, *PRKARIA* and *PRKARIB*) in GBM samples (*n* = 540). Data were interrogated using the r2 platform (https://hgserver1.amc.nl/cgi-bin/r2/main.cgi) to obtain Kaplan–Meier plots that were thresholded at the best expression level predictive of survival. Independent validation was obtained by interrogating the REMBRANDT database, which collects data from 178 GBM patients generated with Affymetrix^®^ HG U133 v2.0plus using the BETASTASIS resource (http://www.betastasis.com/glioma/rembrandt/), thresholding at the median value to discriminate high and low expression. The TCGA database was also explored with the University of California Santa Cruz (UCSC) XENA browser (https://xenabrowser.net/), to compare the data from GBM patients to data obtained from normal brains in the GTEX study (*n* = 158), generated with Illumina TrueSeq RNA sequencing and Affymetrix^®^ Human Gene 1.1 ST Expression Array V3. The analysis of survival was performed with chi-square comparing the two groups (high vs. low expression, thresholds as indicated in the text).

### 4.2. Patients

The study was conducted under the 1964 Declaration of Helsinki and its later amendments. It conformed to Italian legislation and was approved by the institutional Ethical committee (*Comitato Etico per la Sperimentazione—Azienda Ospedaliera di Padova* protocol number 1883P). Patients gave their written informed consent for taking the samples (for immunohistochemistry, Western blot and establishing cell lines) and were recruited at the University of Padova Medical School. Twenty-eight patients were examined (see Supplementary Table S1 for details): 11 had GBM (of which 2 patients were examined again at relapse), while the other samples were from 17 patients and were used for comparison. The sample from one patient included a fragment of unaffected temporal cortex (identified via histology and immunohistochemistry) that was used for comparison. During surgery for tumor removal, one fragment of the tumor tissue was excised and used for cultures, and another was immediately frozen after excision by dipping in liquid nitrogen. Tissues were stored at −70 °C until sectioning at 20 μm with a cryostat.

### 4.3. Chemicals

Chemicals were from Sigma (Milan, Italy), unless otherwise stated. Reagents for cell cultures were from Gibco (Milan, Italy).

### 4.4. Cell Cultures and Pharmacological Treatments

Human high-grade glioma cell lines A172 (mutated in *PTEN* and *CDKN2A*), U87 (mutated in *PTEN*, *CDKN2A*, and *CDKN2C*), and LN229 (mutated in *P53*) were obtained from ATCC (LGC, Sesto S. Giovanni, Italy). Human GLI36 glioblastoma cells were obtained from the University of California [44]. Cells were grown and maintained in monolayer in Dulbecco Modified Eagle’s Medium (DMEM), with streptomycin/penicillin/tetracycline (100 μ/mL) and 10% fetal bovine serum, at 37 °C, with 5% CO_2_. Primary cell cultures were obtained from biopsies (see above) and maintained in DMEM supplemented with antibiotics and 10% fetal bovine serum [45]. They were used at the fourth passage for both immunocytochemistry and Western blot. For pharmacological treatments [15], cells were plated on 24-well plates and maintained in DMEM. After 48 h, one of the following substances was added to the culture: 500 μM glutamate (as an apoptosis-inducing drug); 1 μM progesterone; two clinically used antiblastic agents (100 nM paclitaxel, 200 nM vinblastine); and a series of chemicals affecting the intracellular cAMP pathway (10 μM forskolin, 500 μM isobutylmethylxanthine (IBMX), 100 μM theophylline, 2 mM caffeine, 500 μM 8Br-cAMP, 500 μM 6-dibutiryl-cAMP, and 12 μM H89) and incubated for 24 h. Mouse glioblastoma GL261 cells were used as positive controls and for comparison with previous data from our laboratory [15] and were obtained at the National Cancer Institute [46,47].

### 4.5. Cell Death Count

Cells were fixed with methanol 24 h after the pharmacological treatments and stained with Wright’s stain. Four slides were used for each pharmacological treatment. A total of 2400 cells were counted, and the percentage of dead cells was calculated for each slide by summing apoptotic and necrotic cells. Apoptosis was determined by morphological criteria as a reduction in cell volume and condensation of chromatin or cell membrane blebbing. Necrosis was determined by increased cell volume, release of cytosolic constituents, and collapse of cell structure. *t*-test/monovariate ANOVA was used to test difference in the percentage of dead cells between control and treated cells.

### 4.6. Western Blots

Cells were scraped and resuspended in 40 volumes of PBS (10 mM phosphate, 150 mM NaCl, 1 mM EDTA, pH 7.6) with protease inhibitor cocktail (Roche, Milan, Italy) and forced 40 times through a 27-gauge needle. They were then centrifuged for 15 min at 6000 rpm at room temperature. The supernatant was collected, and the pellet was washed again with the same volume of PBS and centrifuged as above twice. The second and third supernatants were discarded and the pellet resuspended in the same volume of PBS. The protein extraction from the 30 mg biopsy was performed as above in 40 volumes of PBS. Pellets and the first supernatant were run on a 12% polyacrilamide gel, then blotted on nitrocellulose membrane, blocked for 60 min with 1.5% bovine serum albumin, and incubated overnight with primary antibodies against: PKA catalytic subunit, PKA RIIA subunit, or PKA RIB subunit (made in rabbit, Santa Cruz Biotechnology, DBA Segrate, Italy, 1:5000 in 1.5% bovine serum albumin), CD133 (made in mouse, Miltenyi, Calderara di Reno, Italy 1:10,000 in 1.5% bovine serum albumin) or nestin (made in goat, Santa Cruz Biotechnology, 1:10,000 in 1.5% bovine serum albumin), incubated for 2 h with horseradish peroxidase (HRP)-conjugated antirabbit secondary antibody (Sigma, Milan, Italy, 1:10,000) and revealed through chemiluminescence (Advanced ECL, Amersham, Milan, Italy). 

### 4.7. Immunocytochemistry and Immunohistochemistry

Cells were grown in coverslips, washed in PBS, and frozen. After thawing, they were incubated for 5 min in 2% Triton X-100 in PBS at room temperature and fixed in 5% formalin and 1% Triton X-100 for one minute at 37 °C. Tissues were cut on a cryostat at 20 μm. Slides were air dried and post-fixed for one hour in 5% formalin at 18 °C, followed by 30 min in 2% Triton X-100 in PBS. Primary antibodies were incubated overnight. The following antibodies were used [15]: RIIA (rabbit, Chemicon, Temecula, CA, USA) and RIIB (rabbit, Chemicon Temecula, CA, USA) both 1:100 on cells, RIIA (rabbit, Santa Cruz Biotechnology) 1:200 on brain slices, 1:500 on cells; RIB (rabbit, Santa Cruz Biotechnology) 1:200 on slices, 1:500 on cells; glial fibrillary acidic protein (GFAP) 1:200 on slices, 1:500 on cells (mouse, Sigma, Milan, Italy); neurofilament 200, 1:200 on slices, 1:500 on cells (mouse, Sigma, Milan, Italy); and Golgin97 1:200 on slices, 1:500 on cells (mouse, Molecular Probes, Milan, Italy). Secondary antibodies (1:200 on tissue, 1:500 on cells) were incubated for 30 min at 37 °C: anti-rabbit IgG Alexafluor 594-conjugate (Molecular Probes-Invitrogen, Milan, Italy), anti-rabbit IgG fluorescein-conjugate (Sigma, Milan, Italy), and antimouse IgG fluorescein-conjugate (Sigma, Milan, Italy). Cell nuclei were counterstained with bis-benzimide (Sigma, Milan, Italy) or DRAQ5 (Cell Signaling, Milan, Italy). Positive and negative controls were always included: positive controls were mouse brain sections incubated with the above antibodies, whose pattern of labeling is known [12,13,14]; negative controls were reacted omitting the primary antibody or incubated with normal rabbit serum and normal mouse serum. After immunohistochemistry, sections were counterstained with hematoxylin–eosin to confirm tumor presence. Slides were analyzed with a Leica epifluorescence microscope (20×, 40×, and 100× objectives) or a Leica confocal microscope (20× or 63× objectives). Images were acquired with the resident software at 782 × 582 pixels with a color digital camera, using the same parameters within each experiment for a conventional microscope, or with the resident software for the confocal microscope. Contrast was enhanced by a maximum of 10% when necessary with Corel Photo Paint12; final figures were prepared using Corel Draw 12.

## 5. Conclusions

The present data suggest that the intracellular distribution of the different PKA isoforms is characteristic of each cell type. PKA distribution varies during physiological modifications and/or pathological transformations, and could be exploited as a diagnostic or therapeutic target in glioblastoma.

## Supplementary Materials

The following are available online at http://www.mdpi.com/2072-6694/10/1/2/s1. Table S1: Patients; Table S2: summary of results in the various tumors; Figure S1: relative expression of PKA regulatory subunits and survival data; Figure S2: immunocytochemistry on primary cultures; Figure S3: PKA in GBM samples; Figure S4: PKA R2 in different brain tumors; Figure S5: PKA R1 and catalytic subunit in various brain tumors.

## Abbreviations

cAMP	cyclic AMP
PKA	Protein kinase A, cAMP-dependent protein kinase
DMEM	Dulbecco Modified Eagle’s Medium
GFAP	Glial fibrillary acidic protein
IBMX	Isobutylmethylxantine
GBM	Glioblastoma multiforme
R	Regulatory subunits of cAMP dependent protein kinases
