# Supplementary material for: Protein Kinase A Distribution Differentiates Human Glioblastoma from Brain Tissue
Source: Cancers (Basel). 2017 Dec 21;10(1):2. doi: 10.3390/cancers10010002 (PMC5789352; doi:10.3390/cancers10010002)
Supplement: Supplementary file 1 [file cancers-10-00002-s001.pdf]

Table S1. Patients.

CODE	SEX	AGE	DIAGNOSIS
1	M	64	Glioblastoma multiforme (GBM, grade IV WHO)
2	M	11	GBM
3	M	62	GBM
3bis	M	64	GBM (case 3 relapse)
4	F	65	GBM
5	F	59	GBM
5bis	F	60	GBM (case 5 relapse)
6	M	69	GBM
7	M	50	GBM
8	M	73	GBM
9	M	71	GBM
10	M	80	GBM
11	M	56	GBM
12	M	8	Anaplastic ependymoma
13	F	4	Astroblastoma
14	F	12	Giant cell astrocytoma
15	M	6	Anaplastic ependymoma relapse (grade III WHO)
16	F	45	Oligodendroglioma relapse
17	M	40	Cerebellar haemangioblastoma (von Hippel-Lindau disease)
18	F	47	Monro's foramen subependymoma (grade I WHO)
19	M	9	Pilocytic astrocytoma (grade I WHO, type 1 Neurofibromatosis)
20	M	57	Prolactin-secreting pituitary adenoma
21	M	73	Non-secreting pituitary adenoma
22	M	73	Non-Hodgkin lymphoma (grade I/II WHO)
23	F	48	Cavernous hemangioma
24	M	23	Pharmacoresistant epilepsy (hippocampal sclerosis)
25	M	71	Metastasis - clear cell renal carcinoma
25bis	M	71	Case 25, frontobasal cortex
26	M	54	Metastasis - pulmonary squamous cell carcinoma
27	M	63	Metastasis - pulmonary adenocarcinoma
28	M	68	Metastasis - colon adenocarcinoma

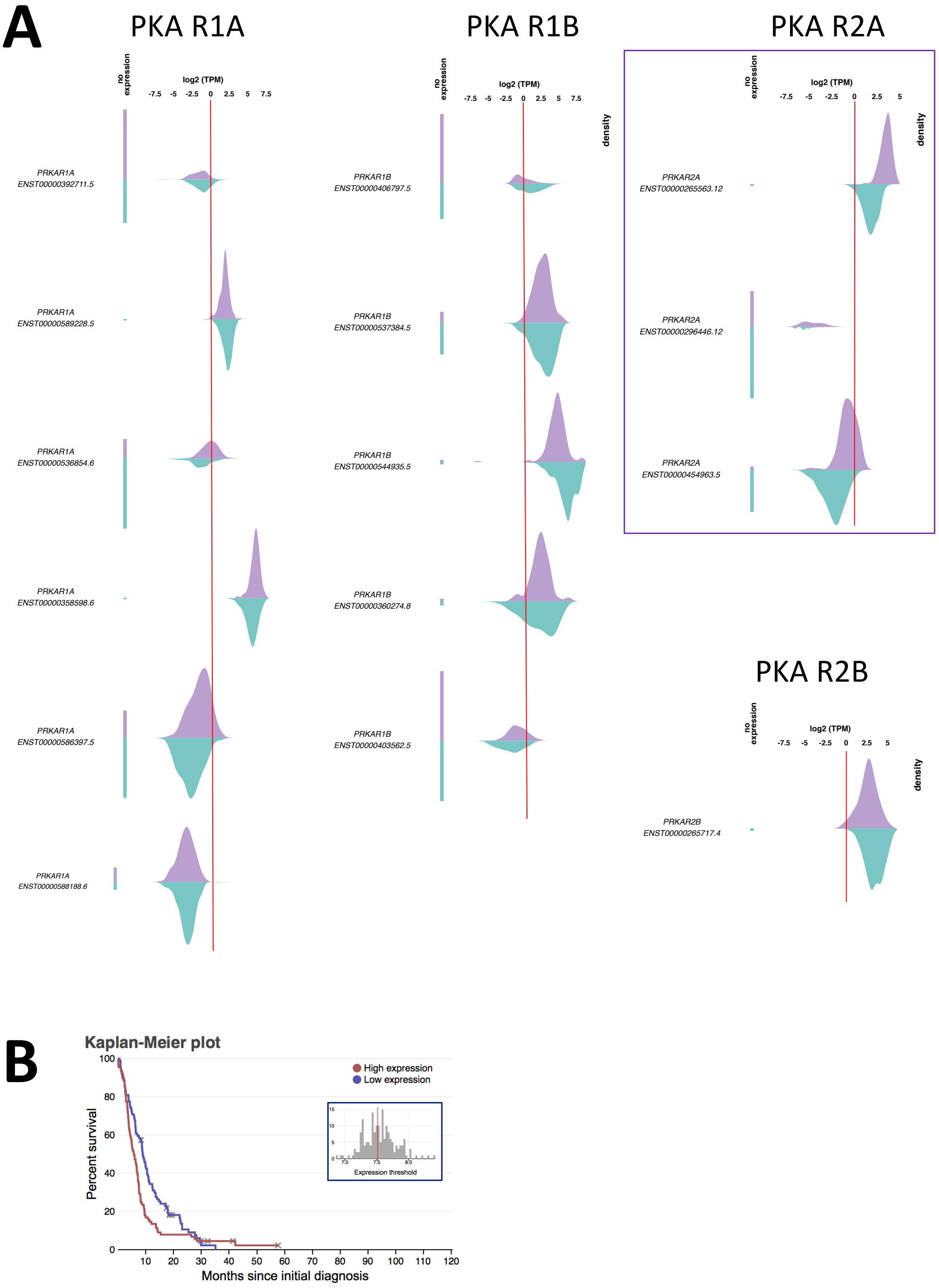

Figure S1 A: The TCGA Glioblastoma Multiforme (n=540) database was comparetd to the GTEX normal brain database (n=158) with the UCSC Xena browser. The level of expression is plotted for each transcript of each gene in purple for TCGA GBM and in green for GTEX brain. The red lines mark the 1 Transcript Per Million landmark. The two major transcripts for PRKAR2A are both more expressed in GBM samples. B: Kaplan-Meier plot, obtained from the REMBRANDT database (n=178), interrogated with the Betastasis thresholded at the median (89 patients in each line, red: high expression, blue: low expression). P<0.01, logranktest.

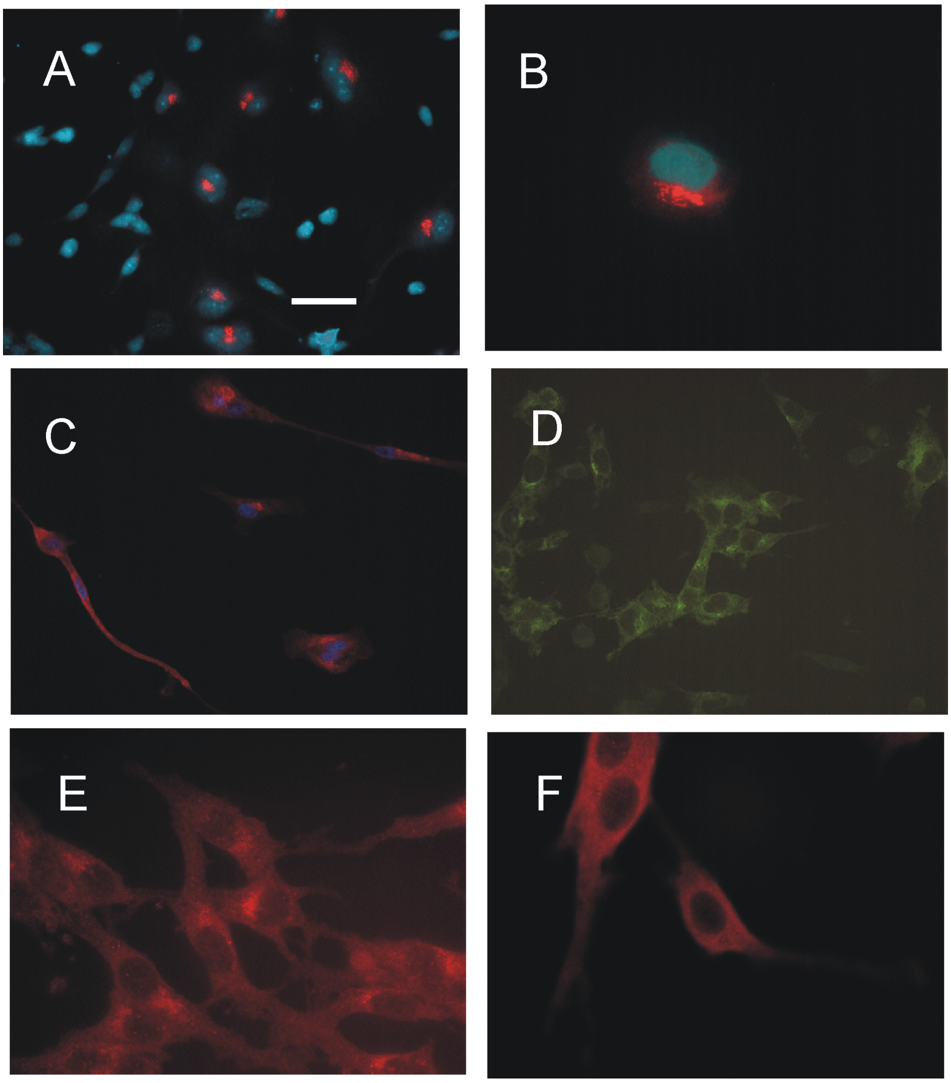

Figure S2. Immunocytochemistry on primary cultures obtained from human brain specimens. (a) R2 (red) is present only in some cells, in a cell culture from normal brain tissue removed to reach the tumor. At variance, in a glioblastoma cell culture, R2 distribution is similar to that in glioma cell line (this figure, b to e). Bis-benzimide (blue) nuclear counterstaining. (b) cell culture from GBM, R2 (red) and bis-benzimide (blue): the similarity with distribution shown in Figure 3 (main text) is apparent. (c) cell culture from GBM case 3, R2 (red) and bis-benzimide (blue). (d) cell culture from GBM case 5, R2 is shown using green-labelled secondary antibodies, to control that its distribution is similar to what obtained with the red-labelled secondary antibody. (e) cell culture from GBM case 6, R2 clustered distribution is shown in red. (f) cell culture from GBM case 6, R1 (red) appears distributed in the whole cytoplasm. Bar = 10 µm (b, e, f); bar = 25 µm (a, c, d).

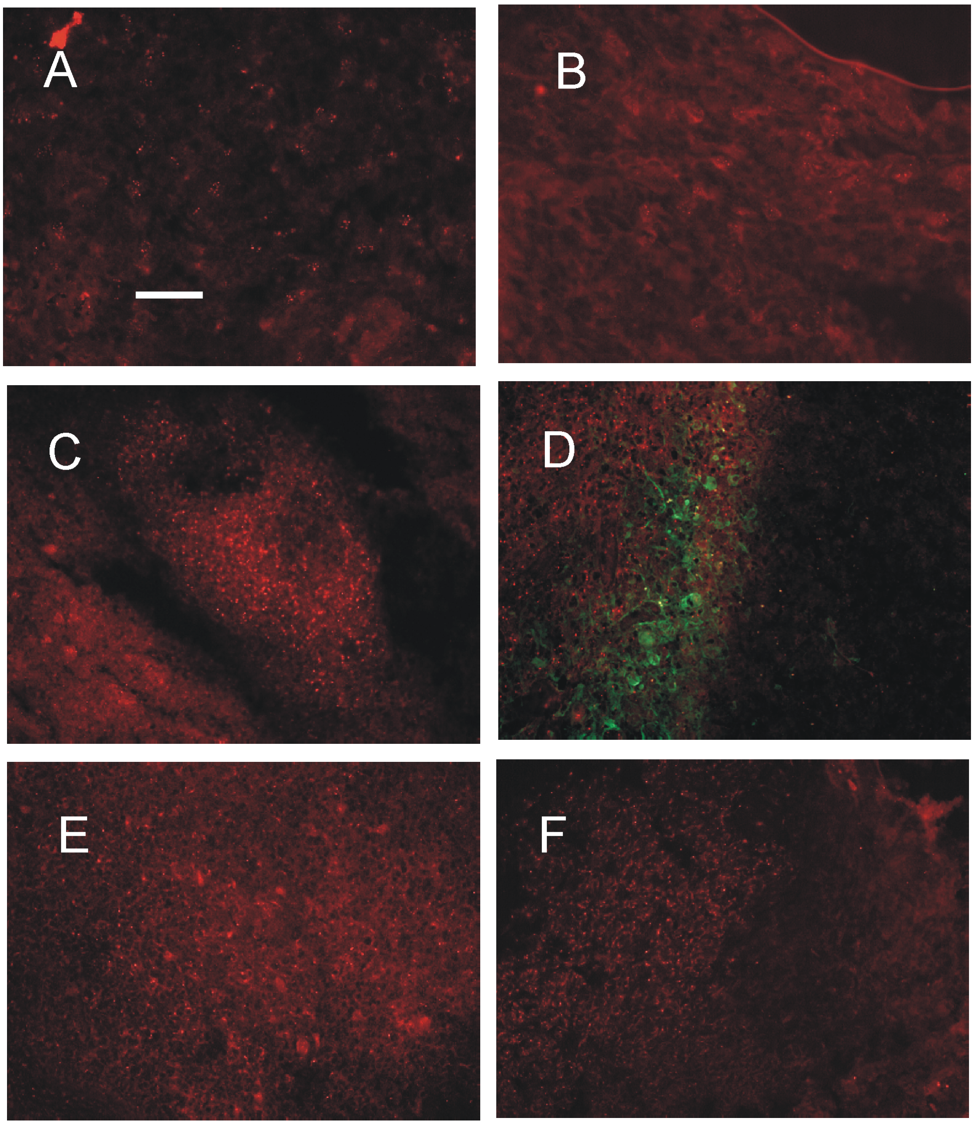

Figure S3. Representative distribution of PKA catalytic and regulatory subunit R1 and R2 in various glioblastoma samples. (a) R1 (red), case 2: clusters of small dots are present only in some areas. (b) R1 (red), case 6: only some cells show a dotted R1 labelling; some cells show also a diffuse cytoplasmic labelling. (c) catalytic subunit (red), case 2: several isolated dots are present in some areas of the tumor. (d) R2 (red) and GFAP (green), case 4: R2 is present on the tumor tissue (left side of the image), while GFAP labels its border. (e) R2 (red), case 2: R2 is present throughout the tumor tissue. (f) R2 (red) depicts the border of the tumor tissue, case 5. Bar = 25 µm (a, b); bar = 50 µm (c, d, e, f).

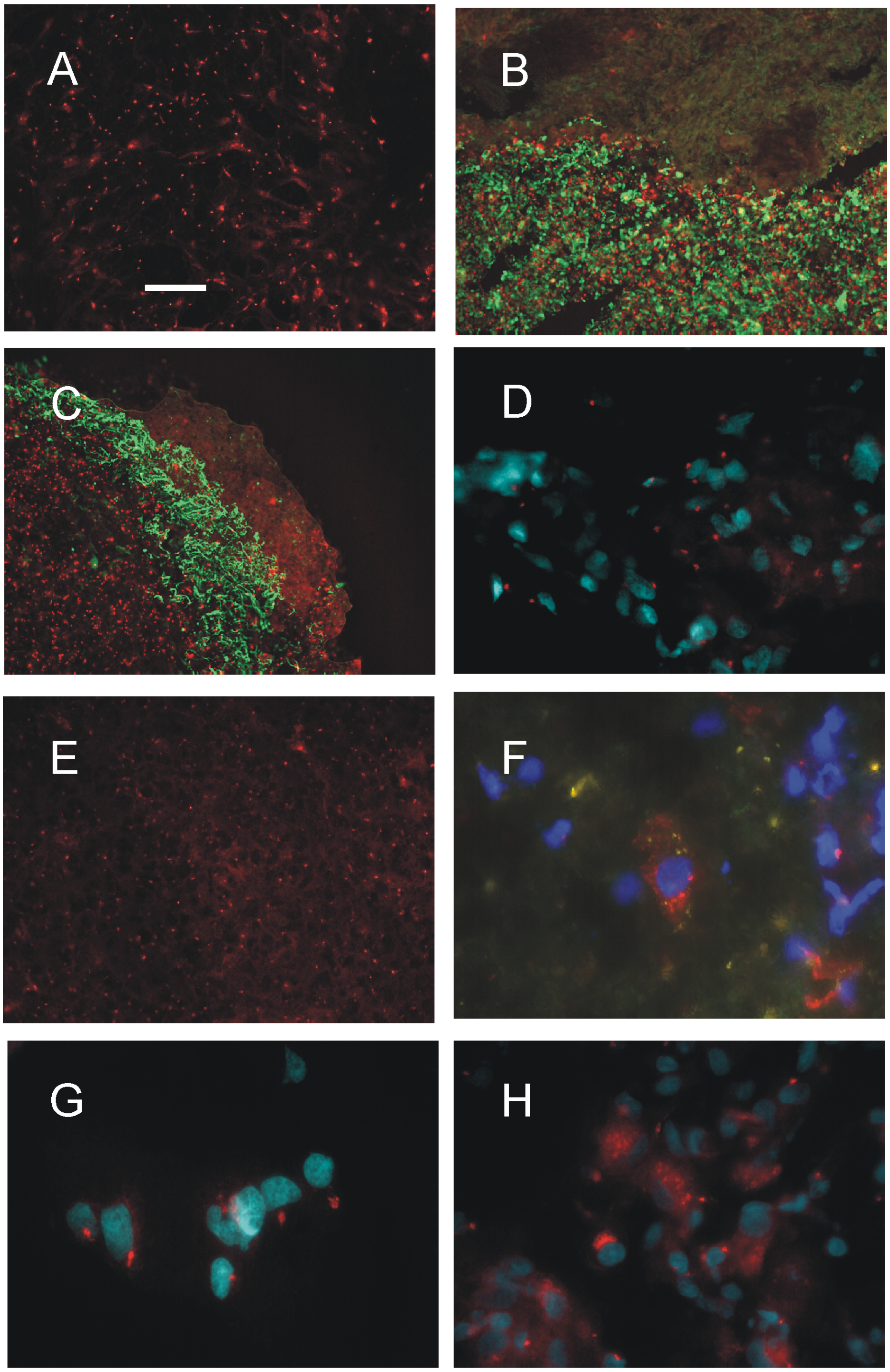

Figure S4. Representative distribution of PKA R2 subunit (red) in various tumors of the brain. (a) case 12, ependymoma: most cells are R2-positive, but R2 distribution appears more variable than in GBM. (b) case 13, astroblastoma: R2 (red) and GFAP (green) show overlapping distribution, albeit non-coincident. (c) case 13, R2 (red) and Neurofilament 200 (green): NF200 appears restricted to the tumor border. (d) case 15, anaplastic ependymoma: R2 (red) and bis-benzimide (blue) show that only some cells, in a few areas, are R2-positive. (e) case 16, oligodendroglioma: only few areas are labelled, like the one showed here, in which some cells are R2-positive. (f) case 17, haemangioblastoma: R2 (red) and bis-benzimide (blue), autofluorescence in yellow: R2 labels only very few cells in the whole section. (g) case 18, subependymoma, R2 (red) and bis-benzimide (blue): only some cells are viable, in which R2 is differently distributed. (h) case 19, astrocytoma, R2 (red) and bis-benzimide (blue): R2 is variously distributed in most of the cells. Bar = 10 μm (d, f, g, h); bar = 25 μm (a, e); bar = 50 μm (b, c).

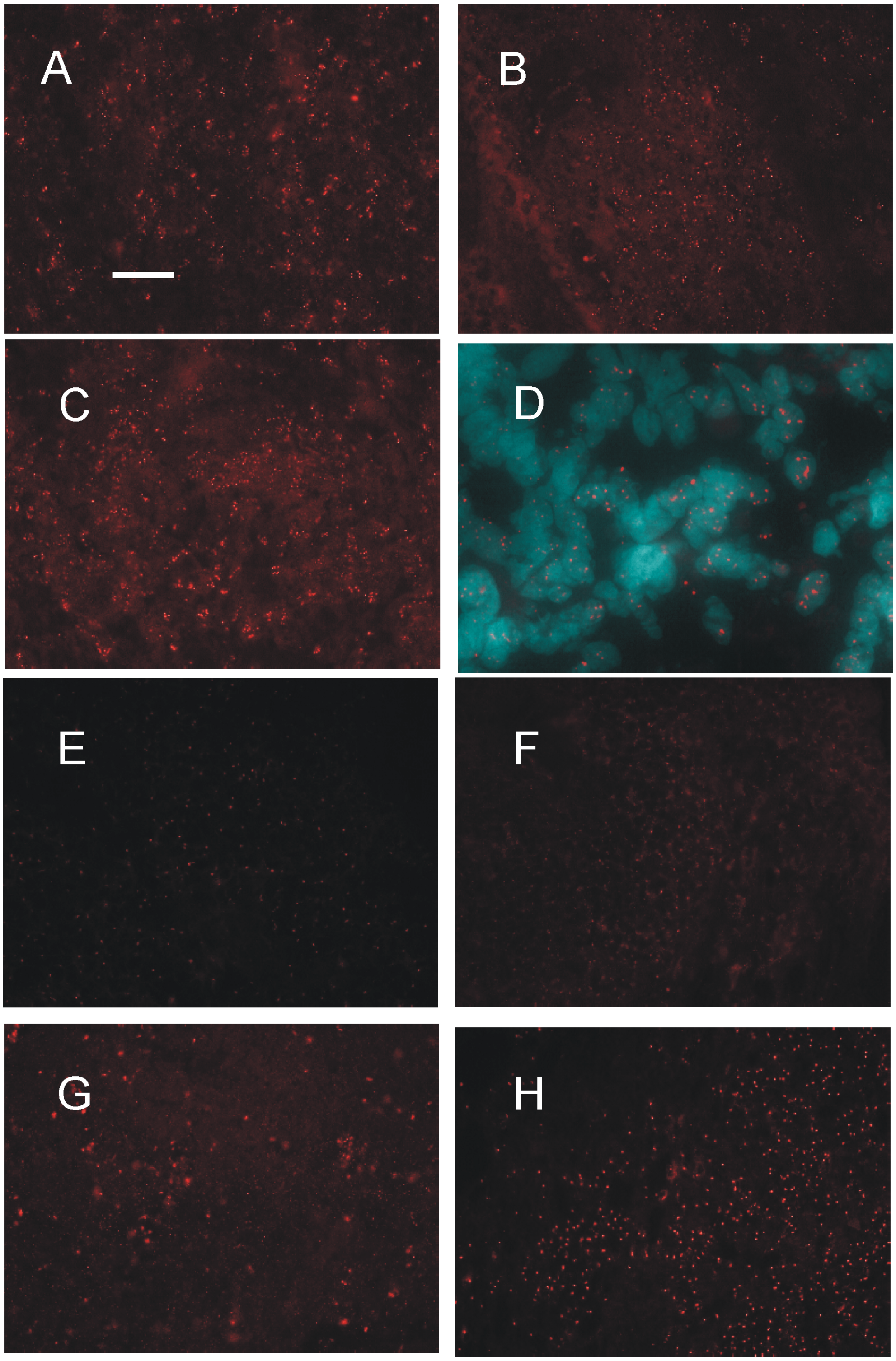

Figure S5. Representative distribution of PKA catalytic and regulatory subunit R1 in various tumors of the brain. (a) R1 subunit (red), case 13, astroblastoma: R1 appears as dots of variable size, present in few areas of the tumor. (b) R1 subunit (red), case 14, astrocytoma: R1 is present in small dots in the tumor area. (c) R1 subunit (red), case 19, astrocytoma: R1 is present as small numerous dots, mostly on the tumor border. (d) R1 subunit (red), bis-benzimide (blue) case 19: nuclear counterstaining shows that R1 dots are variably distributed in only some cells. (e) catalytic subunit (red), case 12, ependymoma: a faint labelling is present in form of isolated dots. (f) catalytic subunit (red), case 15, ependymoma: the labelling is barely detectable. (g) catalytic subunit (red), case 17, haemangioblastoma: the catalytic subunit is present as numerous faint dots and in some clusters. (h) catalytic subunit (red), case 18, subependymoma: the catalytic subunit is present in dots, that at higher magnification present as small circles. Bar = 10 µm (d); bar = 25 µm (a, b, c, e, f, g); bar = 50 µm (h).

Table S2. Summary of immunohistochemical data on PKA regulatory and catalytic subunits.

Case	Diagnosis*	R2	R1	CATALYTIC
**1**	GBM	+	n.a.	n.a.
**2**	GBM	+	small dots in some areas	Some dots only
**3**	GBM	+	small dots, in the cytoplasm	various shapes
**4**	GBM	+	rare dots	-
**5**	GBM	+	rare dots	rare dots
**6**	GBM	+	small dots, in the cytoplasm	rare dots
**7**	GBM	+	-	rare dots°
**8**	GBM	+	rare dots	rare dots°
**9**	GBM	+	rare dots	many dots
**10**	GBM	+	-	in some areas
**11**	GBM	+	-	in some areas
**12**	Ependymoma	+	rare dots	rare dots
**13**	Astroblastoma	+	rare dots	small dots
**14**	Astrocytoma	in some areas	small dots	only in 1 area
**15**	Ependymoma	in some areas	rare brilliant dots	barely detectable°
**16**	Oligodendroglioma	in some areas	-	very few in 1 area°
**17**	Haemangioblastoma	+	-	very faint dots°
**18**	Subependymoma	different shapes	-	small circles
**19**	Astrocytoma	different shapes	small sots on borders	rare dots
**20**	Adenoma	differnt shapes	rare dots	rare faint dots°
**21**	Adenoma	different shapes	rare dots	-
**22**	Lymphoma	few isolated dots	rare dots	-
**23**	Hemangioma	very rare dots	-	-
**24**	Epilepsy	different shapes	few dotted cells	-
**25**	Metastasis	-	-	-
**25bis**	P25 Cortex	rare dots	pyramidal neurons	-
**26**	Metastasis	rare dots	rare dots	many isolated dots
**27**	Metastasis	different shapes	-	in some areas
**28**	Metastasis	-	-	-

* For diagnosis, see Table S1

n.a.: not applicable: some conditions were not tried due to tissue limitations.

-: negative in repeated tests

°: faint labelling in some areas only, in most repetitions of the experiment. In some repetitions, the labelling was not present.
